# Symptoms and distress across the menstrual cycle in a representative sample of Austrian and German people who menstruate: A cross-sectional study

**DOI:** 10.1177/17455057261462827

**Published:** 2026-07-16

**Authors:** Lucia Volpi, Laura M. König

**Affiliations:** 1 Faculty of Psychology, 27258 University of Vienna, Vienna, Austria

**Keywords:** menstrual symptoms, menstrual pain, premenstrual phase, menstrual phase, intermenstrual phase

## Abstract

**Background:**

People who menstruate may experience a range of symptoms, which, fluctuating throughout the different cycle phases, can interfere with their normal functioning. To date, research mostly investigated pain and psychological changes in the menstrual and premenstrual phases.

**Objectives:**

This survey aimed to broaden the understanding of the frequency and interference with daily living (i.e., distress) of a wider array of symptoms (e.g., gastrointestinal, physiological) throughout the cycle, including the intermenstrual phase.

**Design:**

Cross-sectional online survey of representative samples (in terms of education, gender and age).

**Methods:**

Menstruating participants (*N* = 336) from Germany and Austria reported the frequency and distress of 25 symptoms occurring in the three cycle phases and rated how often the symptoms impaired their daily functioning.

**Results:**

The symptom distress remained modest across all phases. Nevertheless, a repeated-measures ANOVA showed that distress was higher during menstruation (*M* = 1.41, *SD* = 0.62) compared to the other two phases (*ps* <.001), *F* (1.79, 526.15) = 91.19, *p* < .001, η^2^_p_ = .24. The psychological-cognitive, discomfort and physiological symptom clusters were the most frequent across all phases. Compared to participants without gynaecological conditions, individuals with a self-reported diagnosis of endometriosis exhibited significantly higher discomfort, pain and impairment across specific cycle phases (i.e., across all phases, during the premenstrual and intermenstrual phases, during the menstrual and premenstrual phases, respectively). Pain predicted impairment of daily functioning in the premenstrual and menstrual phase, together with discomfort. Age, contraception use, and gynaecological conditions were not related to changes in distress.

**Conclusion:**

A broad range of symptoms occurred, going beyond symptoms typically studied, (i.e., discomfort with vaginal bleeding, feeling dirty, concentration impairment). Although with minimal interference, symptoms stereotypically linked to the (pre-)menstrual phase were also reported in other phases. However, given the methodological constraints of this study, this pattern must be interpreted with caution.

## Introduction

The menstrual cycle (MC) is the cyclical growth and decline of the internal lining of the uterus and it is orchestrated by the hormones secreted within the hypothalamic-pituitary-gonadal axis. Beyond executing reproductive functions, it is one of the most important indicators of health in women and people assigned female at birth.^1^ Importantly, the fluctuations of these hormones can lead to a range of symptoms that may impact daily life throughout the menstrual cycle.

The most widely researched symptom in general populations is dysmenorrhea (i.e., abdominal pain related to the MC), followed by other physical occurrences such as heaviness of bleeding, psychological complaints (e.g., anxiety, irritability) and other symptoms (e.g., tiredness, fatigue, headache),.^2–13^ Overall, estimates of the prevalence of dysmenorrhea alone varies greatly; surveys report prevalences between 15%^4^ and 85%.^2^

Although symptoms occurrence, frequency and interference are part of diagnostic criteria for different gynaecological diagnosis such as premenstrual syndrome (PMS) and premenstrual dysphoric disorder (PMDD), they can contribute to limit functionality even without a medical diagnosis. For instance, Schoep et al. (2019) found that in a sample of over 40.000 Dutch women, 38% were unable to perform all their regular daily activities when menstruating.^2^ Likewise, premenstrual physical and mental symptoms may negatively affect activities of daily life, as found in the large cross-cultural study.^10^

Most studies investigating the prevalence of MC symptoms and/or their impact on daily life in general populations have focused on either the menstrual phases ^2[Dutch], 3[Scotland], 5[France], 6[Spain], 7[Hungary], 9[Australia], 11[USA], 12[Brazil], 13[USA], 14[Germany]^ or premenstrual phase ^10[France, Germany, Hungary, Italy, Spain, UK, Brazil, Mexico, Hong Kong, Pakistan and Thailand], 11[USA], 15[Italy]^ with two studies failing to differentiate between phases ^4[Japan], 8[Austria]^.

This overemphasis on a limited number of symptoms researched only in the pre- and menstrual phases^2,6,9^ heavily limits the understanding of the impact and frequency of symptoms across multiple menstrual phases and fails to capture the bread of experiences across the cycle. Indeed, other symptoms, such as physiological, cognitive and gastrointestinal, may also have an impact on menstruators’ lives.^16,17^ Additionally, the need to investigate a broader range of symptoms stems from evidence indicating that different symptom clusters can interact and influence one another. For example, individuals with depressive or anxious symptoms may be more likely to report multiple gastrointestinal symptoms in both menstrual and premenstrual phases.^16^

The evaluation of functional interference caused by menstrual symptoms within general populations is limited by the lack of use of validated assessment tools. Existing studies predominantly focus on the subjective impact of only *pain and bleeding* on daily life,^3,5–7,9^ and operationalise impairment as the extent to which such symptoms are perceived as “ a bother”,^9,11^ “a problem”^3^ or rely on general disability assessment tools.^5^ The few studies to date which have examined the functional interference caused by a *broader* spectrum of menstrual symptoms have significant methodological limitations. Specifically, they did not employ validated instruments^2,10^ or failed to report the interference of individual symptoms, focusing solely on the *overall* impact of symptoms.^8^ This highlights a significant gap in the literature and stresses the need for more comprehensive and standardized approaches to investigating the impact of menstrual symptoms on daily functioning, particularly given that menstrual cycle impairment may persist for roughly 10-15 days per month, when menstrual and premenstrual days are considered together.

Moreover, while there is evidence for premenstrual and menstrual interference,^2,10^ there is limited knowledge about menstrual symptoms occurrence and impairment in phases of the cycle other than the bleeding and premenstrual stages. In this sense, the so called “Mittelschmerz” or ovulation pain may affect over 40% of women of reproductive age, ranging from a mild ache to intense pain.^18^ Besides, ovulation discharges have mostly been scientifically investigated in the context of fertility estimation^19,20^ but not as much in terms of distress and discomfort. The limited scientific data on ovulation discomfort and other ovulation-related symptoms may stem from a cultural reluctance to acknowledge potential negative aspects of reproduction, historically regarded as the primary purpose of female bodies. This gap has resulted in a very shallow understanding of how menstrual symptoms distress fluctuate across the cycle.

Finally, some studies including representative surveys from Hungary,^7^ the UK,^3^ Japan,^4^ and a 14-country study including data from several European countries,^10^ are already more than 10 years old. Given that the public discourse on menstruation and related symptoms changed considerably over time – amongst others, through social media^21^ – it could be assumed that more individuals are becoming aware of their symptoms and related distress or feel more comfortable disclosing it.

The lack of exhaustive and comprehensive menstrual symptoms assessment is also evident in both the latest Austrian^8^ and German menstrual health reports^14,22^ which investigated intensity of bleeding and pain as main outcomes, with other symptoms’ prevalence and interference being investigated without differentiating between premenstrual or menstrual phases^8^ or validated assessment scales.^14^ This study closes this important data gap in both countries and reports on a pre-registered cross-sectional survey conducted in Austria and Germany.

First, it explores the prevalence of a wide array of menstrual symptoms (individual and symptoms clusters) in the menstrual and premenstrual phases, compared to the remaining days of the cycle (research question [RQ] 1). Second, it evaluates the distress of these menstrual symptoms (i.e., interference with daily living) across three stages of the cycle (i.e., menstrual, premenstrual and intermenstrual) using a validated measure (RQ2). Third, considering evidence suggesting an age-related effect on the experience of symptoms,^4,10,12,23,24^ the study explores age as a potential factor influencing menstrual symptoms distress (RQ3). Lastly, it investigates how different symptom clusters contribute to symptoms impairment, operationalised as the frequency of the interference with daily living (RQ4).

## Method

Reporting follows the STROBE guidelines for cross sectional studies.^25^

### Sample

Participants were recruited in May 2024 as part of a larger study investigating digital health.^26^ It sampled 504 Austrian and 1006 German adults (i.e., aged 18 years and older). Recruitment was conducted through a panel provider who compensated participants according to their remuneration policy. For the present analysis, participants were included if they had female reproductive organs (Out of the 336 participants, *N* = 51 reported having menstruated at least once in the past year but did not select the option ‘I have a uterus.’ While it is possible that some of these individuals underwent a hysterectomy within the past year, others may have misclicked or been unaware of what a uterus is. All of them were nevertheless included in the analysis) and menstruated at least once in the previous 12 months. Based on these criteria, *N* = 336 could be included, comprising *n* = 134 individuals from Austria (40%) and *n* = 202 individuals from Germany (60%). We aimed to reduce potential sources of bias by recruiting a relatively representative sample and by avoiding explicit mention of menstruation or the study’s core research questions in the study description, thereby minimising self-selection and response bias.

### Procedure

Ethical approval was granted by the University of Vienna Ethics Committee (approval number 01141). Data was collected online. Participants were informed about the study procedure before providing informed consent by ticking a box. They then completed a range of measures assessing demographic information, personality traits, nutrition- and fitness-related app usage, and menstrual cycle-related variables. Participants were debriefed after the completion of the survey and re-directed to the panel provider for payment.

### Measures

The original questionnaires and an English translation are available from the respective Open Science Framework (OSF) page https://osf.io/2zbfy and in the supplementary material.

#### Sociodemographic information

Sociodemographic information included age, gender identity, sexual orientation, country, rural vs. urban residency, citizenship, immigration background, minority status, highest school leaving certification, highest educational qualification, NET household monthly income, and employment status.

#### Biological sex

Biological sex was assessed via the questionnaire of Peters and colleagues.^27^ Response options included biological sex (i.e., female: born with female primary sexual organs [vagina, ovaries/ovary, uterus], entry “female” on the birth certificate; male: born with male primary sexual organs [testicles], entry “male” on the birth certificate; intersex: born with primary sexual organs whose combination cannot be clearly categorized as female or male; other) and reproductive organs (e.g., “I have at least one ovary”/“I have at least one testicle”).

#### Gynaecological characteristics

Gynaecological characteristics included: contraceptives usage (i.e., pill, contraceptive/hormonal patches, vaginal ring, contraceptive injection, implant/contraceptive rod, hormonal coil, other, none), diagnosis of gynaecological conditions (i.e., premenstrual syndrome PMS, premenstrual dysphoric disorder PMDD, endometriosis, other, none), and medications use to treat gynaecological conditions (i.e., non-steroidal anti-inflammatory drugs, antidepressants/selective serotonin reuptake inhibitors, hormonal preparations, other, yes but do not remember name, none). Hormonal contraceptive use and diagnosed gynaecological conditions were recorded into binary variables (i.e., 1 = yes, if at least one gynaecological condition is indicated; 0 = no, absence of diagnosis of gynaecological conditions). Diagnosis of gynaecological conditions were self-reported, and no information was collected on how these diagnoses were established.

#### Symptoms and distress

Twenty-five symptoms and related distress were measured by the Menstrual Distress Questionnaire – MEDI-Q.^17^ Deviating from the original scale, participants were asked about the symptoms distress in all the three stages (i.e., menstrual, premenstrual and intermenstrual) regardless of the symptoms’ occurrence in the menstrual phase (as designed in the original version of the MEDI-Q). This adaptation was implemented to have an estimation of the occurrence of symptoms across the entirety of the cycle without using the menstrual phase as benchmark, aiming to potentially avoid missing out on symptom distress that is only present in the pre- or intermenstrual but not the menstrual phase.

#### Impairment of daily living activities

For the premenstrual and menstrual phases separately, impairment of daily living activities was measured with a self-developed single item on a 7-point-Likert scale: 1 = never to 7 = always (“In the past 12 months, how often have the symptoms caused by menstruation been so severe that they have prevented you from carrying out your normal daily activities?”).

### Data analysis

The data analyses plan was preregistered on OSF: https://osf.io/8jfmk. Analyses were performed with SPSS 29. Deviations from the preregistration are described below.

#### Calculation of menstrual distress scores

The survey utilised a forced-entry design, and the only missing data in this dataset were classified as logical missingness. Participants who selected “symptom is not occurring” in Score A (frequency of symptoms during the menstrual phase) were *not* asked to rate the distress caused by symptoms (Score B), which response was coded as logical missingness. For Scores C and D, responses with a value of 5, indicating “symptom is not occurring,” were recoded as logical missing to ensure alignment with the coding approach used for Score B.

Menstrual distress scores and subscales were computed following the procedure by Vannuccini and colleagues.^17^ Specifically, a 4-point Likert scale, ranging from “0 = no distress”, to “3 = severe distress”, assessed symptom distress in three phases of the cycle (i.e., menstrual phase = score B; premenstrual phase = score C; intermenstrual phase = score D). The frequency of symptoms in the menstrual phase was measured differently compared to the other two phases: namely on a 3-point scale, ranging from “0 = symptom has never occurred” to “2= this symptom occurred during at least half of the menstrual periods in the last 12 months”).

Instead of using the pre-registered cumulative frequency calculation (which was omitted to aid in result interpretation), two types of frequency were calculated.

1. Absolute frequency: calculated over the total sample size for individual symptoms (i.e., *N* = 336) and over the sample size* the total number of MEDIQ items (i.e., *N* = 8400, 336*25) for clusters.
2. Relative frequency: calculated over the number of participants*number of items of the cluster. This was done to account for the different number of cluster items.

Table 1 presents the four scores calculated following the scoring recommendations of the original scale^17^ (see also https://osf.io/zcya4). Adding to the pre-registered subscales, we calculated, exploratorily, four additional scores representing the total number of symptoms increasing in distress in the intermenstrual and premenstrual phases compared to the menstrual phase, and their related average distress. This decision stems from the original scoring of the MEDI-Q not taking into consideration those symptoms that may cause more distress during other phases of the cycle than the menstrual, which is central to our research questions. Furthermore, deviating from the original scoring of the premenstrual and intermenstrual symptoms distress – which codes *both* the absence of distress and the absence of symptoms as 0 – we included *only* the absence of distress in the distress calculation (subscales and distress average). This decision was made to align the scoring of the premenstrual and intermenstrual distress with that of menstrual distress, which distinguishes symptom frequency (absence or presence) from symptom distress.

**Table 1.** Scores calculated based on the MEDI-Q and exploratory scores.

Score	Interpretation	Calculation
Scores calculated following MEDI-Q original scale^17^		
Total Score	Symptoms distress experienced in the menstrual phase (difference between score B and D), adjusted for frequency (score A). Values ≥ 20 indicate a clinically relevant condition.	Total Score=∑((Score B−Score D)×Score A) *
Menstrual symptoms (MS)	Number of symptoms that generate more distress during menstruation than in intermenstrual days	MS=∑(Total Score>0)
Menstrual symptoms distress (MSD)	Average distress related only to those menstrual symptoms that have been reported to exacerbate during menstruation compared with intermenstrual days	$MSD=\frac{\text{Total}\;\text{score}}{\text{MS}}$
Menstrual Specificity Index (MESI)	Proportion of symptoms for which the participant has reported an exacerbation of distress relating to intermenstrual and premenstrual days. Range between 0 and 1 where: “0 = all the symptoms experience during menstruation are present at least with the same level of distress in the premenstrual phase” and “1 = all the reported symptoms experience more distress during menstruation compared with the premenstrual phase”.	​
		$\text{MESI}=\frac{\sum \left(\left(\text{ScoreB}\;>\text{Score}\;D\right)\wedge \left(\text{Score}\;B>\text{Score}\;C\right)\right)}{\text{MS}}$
Additional exploratory scores		
Intermenstrual symptoms score (IS)	Number of symptoms that generate more distress during intermenstrual than in menstrual days	IS=∑(Score D>Score B)
Intermenstrual symptoms distress score (ISD)	Average distress related only to those menstrual symptoms that have been reported to exacerbate during intermenstrual days compared with menstrual days	$\text{ISD}=\frac{\text{Average}\;\text{intermenstrual}\;\text{distress}}{\text{IS}}$
Premenstrual symptoms (PS)	Number of symptoms that generate more distress during premenstrual than in menstrual days	PS=∑(Score C>Score B)
Premenstrual symptoms distress (PSD)	Average distress related only to those menstrual symptoms that have been reported to exacerbate during premenstrual days compared with menstrual days	$\text{PSD}=\frac{\text{Average}\;\text{premenstrual}\;\text{distress}}{\text{PS}}$

*Note.* ∑=sum ; ∧=and ; Score A = frequency of menstrual symptoms in the menstrual phase. Score B = individual symptoms distress in menstrual phase; Score C = individual symptoms distress in premenstrual phase; Score D = individual symptoms distress in the intermenstrual phase; Average intermenstrual distress = average distress computed cross all the symptoms in the intermenstrual phase. Average premenstrual distress = average distress computed cross all the symptoms in the premenstrual phase.

*If the result of the multiplication is equal to 6 it was replaced by a score of 5 instead according to Vannuccini and colleagues.^17^

To test the difference of average distress across menstrual cycle phase, the means of distress scores of all symptoms in the three phases of the cycle were calculated. Cronbach’s alpha of symptoms distress was calculated for each of the menstrual phases: menstrual phase α =.91; intermenstrual α =.98; premenstrual α =.97.

Lastly, average distress scores for each symptoms cluster for every cycle phase were computed. Five clusters were calculated containing the following items.

1. Pain: lower abdominal pain, urinary pain, pain at defecation, muscle or osteoarticular pain, headache, pain during sexual intercourse.
2. Gastrointestinal: constipation, diarrhoea, nausea, digestive problems.
3. Discomfort: breast tenderness or widespread swelling sensation, feeling uncomfortable about vaginal blood loss, feeling of being impure
4. Psychic and cognitive changes: sadness, emotional lability, irritability or anger, impulsiveness, anxiety, concentration impairment.
5. Physiological: increased appetite, decreased appetite, insomnia, hypersomnia, excessive tiredness and decrease libido.

Cronbach’s alpha for each cluster by phase are listed in the supplementary material (Table 1).

#### Statistical analysis

The frequency and average distress of individual symptoms and clusters were calculated to explore the prevalence of symptoms across the cycle (RQ1). Repeated measures ANOVAs were run to test differences across distress scores between different phases of the cycle (RQ2). Assumptions of sphericity were violated, and Greenhouse-Geisser correction was applied (ε = .90). A mixed ANOVA was performed with contraception and gynaecological condition status as between-subjects factors to test their effects on distress changes across menstrual cycle phase investigated in RQ2. Age effects on the difference distress scores were tested using a repeated measure ANCOVA (RQ3). Multiple regression analyses were run between symptoms clusters and the average symptoms impairment to understand which cluster is reported to impact normal functioning the most in the premenstrual and menstrual phases (RQ4). The level of statistical significance was set to α = 0.05 for all analyses. Interpretation of the effect size was based on Cohen’s^28^ for effect sizes (e.g., Cohen’s d for post hoc comparisons and partial η^2^ for the ANOVAs.).

##### Deviation from pre-registered analyses and tables

Two sets of analyses were not part of the preregistered plan and should be regarded as exploratory. First, in response to the reviewer’s request to examine gynaecological comorbidities more closely, we conducted additional independent-samples *t*-tests comparing participants with self-reported endometriosis to those without any reported gynaecological condition on pain, bleeding-related discomfort, and impairment across menstrual, premenstrual, and intermenstrual phases (Table 2 in supplementary material). These tests, together with Cohen’s *d* and 95% CIs, were added as sensitivity analyses and are not used to draw confirmatory conclusions, given the small endometriosis subgroup.

Second, for the tables representing phase-specific symptom frequencies we went beyond the preregistered binary prevalence (symptom present vs. absent). We now report the full distribution of distress ratings on the 0–3 scale (0 = no distress, 1 = somewhat, 2–3 = moderate/severe) plus the proportion of participants indicating that the symptom does not occur. In addition, for the full sample we derived two prevalence thresholds – any distress (score ≥ 1) and moderate–severe distress (score ≥ 2) – and calculated exact binomial 95% confidence intervals and binomial *p* values for these proportions. These additions provide a more nuanced, clinically oriented description of symptom burden but were not preregistered hypothesis tests.

## Results

### Sample characteristics

Table 2 shows the demographic composition of the sample and the menstrual cycle related characteristics. The mean age was 34.6 (*SD* = 10.9). Most of the participants identified as women (97%) and heterosexual (84%). Most of the sample did not have a gynaecological diagnosis (85%). However, of the 15% people diagnosed, the majority had PMS (5% of the full sample), followed by endometriosis (5%), other (e.g., Polycystic ovary syndrome PCOS, 3%), and premenstrual dysphoric disorder PMDD (2%). Two participants marked both PMS and no diagnosis, thus were treated as healthy participants. The pill was the most common hormonal contraception used (24%), and 66% did not use contraception.

**Table 2.** Demographics and gynaecological information.

Sample characteristics	*N*	*%*
Country		
Austria	134	40%
Germany	202	60%
Gender		
Woman	327	97%
Man	8	2%
Other	1	0%
Sexual Orientation		
Heterosexual	282	84%
Other	54	16%
Gynaecological condition		
PMS	15	5%
PMDD	8	2%
Endometriosis	18	5%
Other	10	3%
None	285	85%
Contraception		
Pill	82	24%
Hormonal coil	9	3%
Hormonal patches	5	2%
Vaginal ring	4	1%
Contraceptive injection	5	2%
Implant	4	1%
Other	4	1%
None	223	66%

The majority of the sample lived in a big city (*N* = 113, 34%), held a citizenship of one of the two countries (*N* = 326, 97%), had no migration background (*N* = 273, 81%), did not identify as a minority (*N*= 281, 84%), had a high school leaving certificate (*N* = 116, 35%), completed an apprenticeship (*N* = 94, 28%), an average monthly income between 3.000 and 5.000 Euros (*N* = 102, 30%) and was employed full-time (*N* = 141, 42%). The breakdown of this demographic information per country is available in the supplementary material (Table 3).

### Research question 1: Description and prevalence of symptoms in the menstrual and premenstrual phases

#### Menstrual phase

The breakdown of the absolute frequencies of individual symptoms during the menstrual phase is available in the supplementary material (Table 4–6). Given the high similarity in occurrence between symptoms reported in less than or at least in half of the menses, the frequencies reported in the paragraph below combine these two scores. Abdominal pain (*N* = 285, 85%) had the highest absolute frequency. This was followed by emotional instability (*N* = 223, 66%), irritability or anger (*N* = 220, 65%), fatigue (*N* = 219, 65%), headache (*N* = 214, 64%), and breast tenderness or swelling (*N* = 207, 62%). As for distress level (see Table 3), headache (*M* = 1.76, *SD* = 0.92) was the symptom reported as being most distressful, followed by concentration difficulties (*M* =1.74; *SD* = 0.91), insomnia (*M* = 1.72, *SD* = 0.95), discomfort due to vaginal bleeding (*M*= 1.72, *SD* = 0.95) and abdominal pain (*M* = 1.69, *SD* = 0.94). The symptoms more frequently reported as “never occurring” were pain when urinating (*N* = 282, 84%), pain during sex (*N* = 266, 79%), decreased appetite (N = 259, 77%), pain during bowel movement (*N*= 251, 75%), constipation (*N* = 256, 76%), and concentration difficulties (*N* = 219,65%).

**Table 3.** Phase-specific prevalence of distress of the five most distressing symptoms per phase, with binomial 95% CIs.

Symptom	Distress						Symptom does not occur		If yes, average distress			Any vs. no distress				Moderate-severe vs. No-Mild distress			
​	No distress		Mild		Moderate - severe		​					95%CI				95%CI			
​	*N*	%	*N*	%	*N*	%	*N*	%	*M*	SD	Mode	%	*LL*	*UL*	*p*	*%*	*LL*	*UL*	*p*
**Menstrual phase**																			
Headache	16	5%	74	22%	124	37%	122	36%	1.76	0.92	1	93%	89%	96%	.000**	58%	51%	64%	.024
Fatigue	16	5%	82	24%	121	36%	117	35%	1.75	0.94	1	93%	89%	96%	.000**	55%	49%	62%	.137
Concentration difficulties	9	3%	40	12%	68	20%	219	65%	1.74	0.91	1*	92%	87%	96%	.000**	58%	49%	67%	.096
Discomfort due to vaginal bleeding	13	4%	76	23%	98	21%	149	44%	1.72	0.94	1	93%	89%	96%	.000**	52%	45%	60%	.559
Insomnia	11	3%	44	13%	68	20%	213	63%	1.72	0.95	1	91%	85%	95%	.000**	55%	47%	64%	.279
**Premenstrual phase**																			
Abdominal pain	85	25%	90	27%	116	35%	45	13%	1.32	1.11	1	71%	65%	76%	.000**	40%	34%	46%	.000**
Headache	74	22%	89	26%	103	31%	70	21%	1.32	1.10	1	72%	67%	77%	.000**	39%	33%	45%	.000**
Fatigue	84	25%	78	23%	101	30%	73	22%	1.27	1.12	0	68%	62%	74%	.000**	38%	33%	44%	.000**
Emotional instability	80	24%	95	28%	103	31%	58	17%	1.25	1.05	1	71%	66%	76%	.000**	37%	32%	43%	.000**
Irritability or anger	85	25%	89	26%	94	28%	68	20%	1.21	1.07	1	68%	63%	74%	.000**	35%	30%	41%	.000**
**Intermenstrual phase**																			
Headache	87	26%	72	21%	96	29%	81	24%	1.18	1.07	0	66%	60%	72%	.000**	38%	32%	44%	.000**
Abdominal pain	102	30%	77	23%	90	27%	67	20%	1.13	1.1	0	62%	56%	68%	.000**	34%	28%	39%	.000**
Fatigue	104	31%	71	21%	83	25%	78	23%	1.08	1.10	0	60%	54%	66%	.002	32%	27%	38%	.000**
Emotional instability	101	30%	77	23%	78	23%	80	24%	1.02	1.02	0	61%	55%	66%	.000**	31%	25%	36%	.000**
Increase need for sleep	103	31%	71	21%	70	21%	92	27%	1.02	1.08	0	58%	52%	64%	.018	29%	23%	35%	.000**

*Note.* “No Distress” = symptom is present but rated as not interfering with daily life (interference = 0 on the 0-3 scale); “Mild” = symptom interferes somewhat (interference = 1 on the 0-3 scale); “Moderate - Severe” = symptom interferes moderately or very strongly (interference = 2-3 on the 0-3 scale); “% ” represents the percentage of participant in the total sample (i.e. *N* = 336); *M* = mean; *SD* = standard deviation: Mode: 0 = symptom does not interfere with daily life, 1 = symptoms somewhat interferes with daily life; *M*, *SD*, and Mode describe the interference ratings (0-3) among participants who experienced the symptom; The number of participants reporting symptoms absence (i.e., “Symptom does not occur” in the table) corresponds to logical missingness and was excluded from the calculation of the mean distress scores; “Any vs. no distress” shows the prevalence of any distress (score ≥ 1) with binomial 95% CIs and p values. “Moderate–severe vs. no–mild distress” shows the prevalence of moderate–severe distress (score ≥ 2) with binomial 95% CIs and p values. CI = confidence interval estimated using Likelihood Ratio; *LL* = lower limit; *UL* = upper limit; *p* = p value of binomial tests. * Two modes exist: 1 = mild and 2 = moderate. ** *p* = < .001.

**Figure 1.**
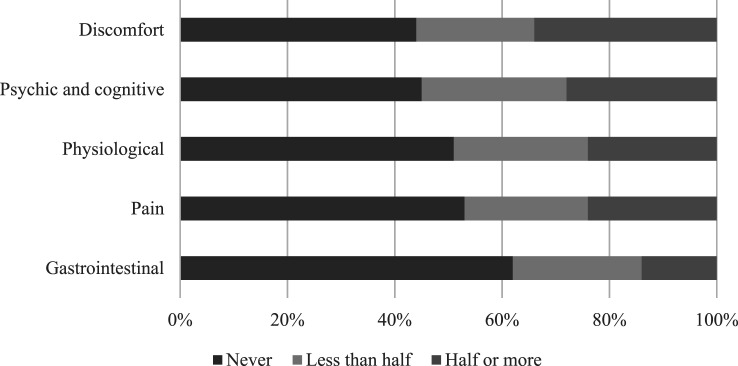
Relative frequency of symptom clusters in the menstrual phase over the past 12 months *Note. “*Never” = symptoms of the cluster haven’t occurred; “Less than half” = symptoms of the cluster have occurred less than half of the menstrual periods in the past 12 months; “Half or more” = symptoms of the cluster have occurred at least during half of the menstrual periods in the last 12 months.

As for symptoms clusters (calculated in terms of relative frequency to account for the number of items in the cluster), the discomfort (57%, *M* = 1.37, *SD* = 0.84), psychic and cognitive (50%, *M* = 1.43, *SD* = 0.79), physiological (46%, *M* = 1.40, *SD* = 0.82) were the most frequent (see Figure 1) but less distressful than pain symptoms (43%, *M* = 1.49, *SD* = 0.76).

#### Premenstrual phase

Table 3 presents the absolute frequencies of the five most distressful symptoms occurring in the premenstrual phase (see Table 7–8 in the supplementary material for a full breakdown of the 25 symptoms). Those which were reported to occur the most in the premenstrual phase were abdominal pain (*N* = 291, 87%), emotional instability (*N* = 278, 83%), irritability or anger (*N* = 268, 80%), headache (*N* = 266, 79%), breast tenderness or swelling (*N* = 259, 77%), and fatigue (*N* = 263, 78%). Abdominal pain (*M* = 1.32, *SD* = 1.11), headache (*M* = 1.32, *SD* = 1.10), emotional instability (*M* = 1.25, *SD* = 1.05), fatigue (*M* = 1.27, *SD* = 1.12), and irritability or anger (*M* = 1.21, *SD* = 1.07) were additionally the symptoms reported as being most distressful. As for symptoms clusters, the psychic and cognitive symptoms (76%, *M* = 1.11, *SD* = 0.91) and discomfort clusters (74%, *M*= 1.05, *SD*= 0.91) were the most frequent and distressful together with the physiological (71%, *M* = 1.06, *SD* = 0.89) and pain clusters (70%, *M* = 1.05, *SD* = 0.85).

#### Intermenstrual phase

Table 3presents the absolute frequencies of the five distressful symptoms occurring in the intermenstrual phase (see Table 9–10 in the supplementary material for a full breakdown of the 25 symptoms). Abdominal pain (*N* = 269, 80%), fatigue (*N* = 258, 77%), emotional instability (*N* = 256, 76%), headache (*N* = 255, 76%), irritability or anger (*N* = 251, 75%), increase need for sleep (*N* = 244, 74%), were the most distressful symptoms reported in the intermenstrual phase. The frequency and distress of symptom cluster in this phase mirrors the one in the premenstrual phase with psychic and cognitive symptoms being the most reported (72%, *M* = 0.97, *SD* = 0.91), followed by discomfort (71%, *M* = 0.97, *SD* = 0.97), and physiological symptoms (69%, *M* = 0.96, *SD* = 0.93).

### Research question 2: Comparison of distress across menstrual cycle phases

#### Comparison based on MEDI-Q scores

**Figure 2.**
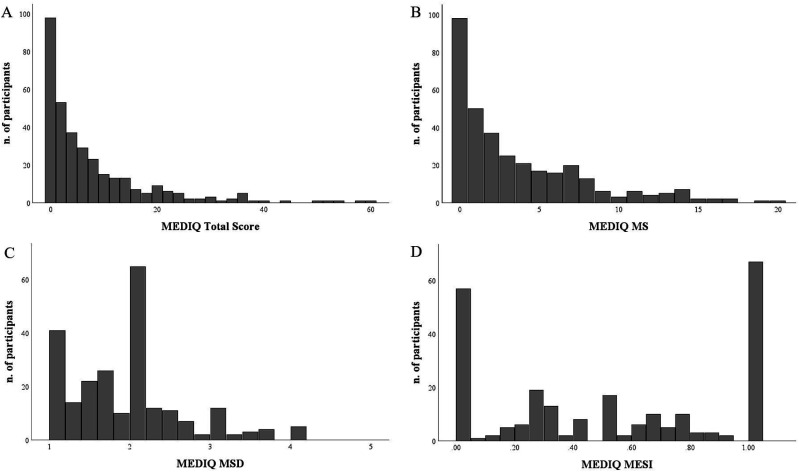
Distribution of MEDIQ scores *Note.* Each panel shows the number of participants for the corresponding MEDIQ score. Panel A: MEDIQ Total Score: overall menstrual distress relative to the intermenstrual phase (frequency-adjusted total score). Panel B: MEDIQ MS presents the number of symptoms that are rated as more distressing in the menstrual than in the intermenstrual phase. Panel C: MEDIQ MSD shows distribution of mean distress of those symptoms that increase from the intermenstrual to the menstrual. Panel D: MEDIQ MESI the proportion of symptoms that are more distressing in the menstrual than in the premenstrual phase (1 = all reported symptoms are more distressing during menstruation; 0 = no symptom is more distressing during menstruation, i.e., all are equal to or less distressing than in the premenstrual phase).

Figure 2(A) presents the MEDI-Q total score, i.e. the sum of those symptoms whose distress score was higher than the intermenstrual phase, adjusting for frequency (*M*_*TS*_ = 7.44, *SD*_*TS*_ = 10.42). Thirty-eight (11%) participants scored was above the clinical cut-off of ≥ 20. A little over 3 symptoms (*M*_*MS*_ = 3.60, *SD*_*MS*_ = 4.18), on average, were recorded to generate more distress during menstruation than intermenstrual days (MEDI-Q MS, Figure 2(B)). However, the average distress of these was relatively low *M*_*MSD*_ = 1.94, *SD*_*MSD*_ =.76, as indicated by the MEDI-Q MSD (Figure 2(C)). Moreover, as the MESI (Figure 2(D)) indicates that the frequency of 1 scores (*N* = 67, 28%) was higher than the frequency of 0 (*N* = 57, 24%). Thus, for most of the sample all the symptoms experienced during menstruation generate more distress compared to the premenstrual phase.

On average, only one symptom was reported to be more distressful in the intermenstrual phase than in the menstrual phase (*M*_IS_ = 1.5, *SD*_*IS*_ = 2.70). However, as shown by the ISD score, the average distress was minimal (*M*_*ISD*_ = 0.65, *SD*_*ISD*_ = 0.56).

Similarly, participants reported an average of approximately two symptoms that caused more distress in the premenstrual than in the menstrual phase (*M*_*PS*_ = 1.82, *SD*_*PS*_ = 2.58), although the overall level of distress was low (*M*_*PSD*_ = 0.60, *SD*_*PSD*_ = 0.55).

#### Statistical comparison of phases

A repeated measures ANOVA examined changes in distress across menstrual phases. The analysis revealed a significant effect of menstrual phase on distress*, F* (1.79, 526.15) = 91.19, *p* < .001, ηp^2^ = .24. Pairwise comparisons with Bonferroni correction indicated a significant reduction in distress from the menstrual phase (*M* =1.41, *SD* = 0.62) to both the intermenstrual (*M* = 0.98, *SD* = 0.81; _95%_CI [-.53, -.34], *p* < .001) and the premenstrual (*M* = 1.06, *SD* =.76; _95%_CI [-.43, -.28], *p* < .001, *d* = 0.64). Both reductions had a medium effect size of *d*= 0.64 ^28^. Distress increased significantly between the intermenstrual and premenstrual phases (_95%_CI [.00, .15], *p* = .03), though the effect size was small d = 0.15;^28^.

A mixed ANOVA tested whether two dichotomous variables — diagnosis and contraception — explained the distress changes across phases. The main effect of phase still reached statistical significance, *F* (1.78, 519.10) = 13.15, *p* < .001, ηp^2^ = .04. Neither diagnosis, *F* (1.78, 519.10) = .43, *p* =.63, ηp^2^ = .00 nor contraception *F* (1.78, 519.10) = 2.32, *p* =.11, ηp^2^ = .01 interacted with phase in predicting distress.

#### Post hoc sensitivity analyses

We conducted non-preregistered independent t-tests comparing pain, discomfort due to vaginal bleeding, and impairment between participants with self-reported endometriosis and those without any gynaecological diagnosis (see Table 2 in supplementary material).

Except for abdominal pain in the menstrual phase, which did not differ significantly between groups (*M* = 2.12 vs. 1.66, *p* = .057, Cohen’s *d* = 0.48), participants with endometriosis showed consistently higher pain, bleeding-related discomfort, and impairment than the comparison group across phases. Effect sizes for these differences were generally in the moderate-to-large range (Cohen’s *d* = 0.69–1.14, all _95%_CIs not including zero); for example, intermenstrual pain was almost one standard deviation higher in the endometriosis group (M = 2.07 vs. 1.02, *d* = 0.98, _95%_CI = [0.43, 1.52]), and discomfort due to vaginal bleeding in the intermenstrual phase exceeded one standard deviation (*M* = 2.00 vs. 0.84, *d* = 1.14, _95%_CI = [0.52, 1.75]). Comparable sensitivity analyses for other common gynaecological comorbidities (e.g., fibroids) were not possible due to the very small number of cases.

### Research question 3: Relationships between distress and age

A repeated-measures ANCOVA was conducted to assess the effect of phase on distress levels with age included as a covariate. Age did not significantly interact with phase, *F* (1.79, 525.43) = 2.01, *p* =.13, ηp^2^ = .01.

### Research question 4: Relationships between symptoms and impairments of daily living activities

Multiple regression analyses tested the relevance of each symptom distress cluster in predicting symptoms impairment to understand which cluster is reported to impact normal functioning more frequently. In the menstrual phase (Table 4), the model explained 34% of the variance in impairment (R^2^ = .34, adj. R^2^ = .32). Pain (β = .28, *p* <.001) and discomfort (β = .22, *p* = .005), were the only two clusters significantly predicting impairment. In the premenstrual phase (Table 5), the model explained 52% of the variance in impairment (R^2^ = .52, adj. R^2^ = .51). Pain (β = .48, *p* <.001) was the only significant predictor.

**Table 4.** Symptoms clusters coefficients predicting symptoms impairment during menstrual phase.

​	95% CI					
Cluster	B	*SE*	*LL*	*UL*	β	*p*
Constant	0.69	0.37	-0.03	1.41	-	.06
Pain	0.81	0.22	0.386	1.234	0.28	.000*
Gastrointestinal	0.23	0.17	-0.106	0.571	0.10	.177
Discomfort	0.49	0.17	0.147	0.832	0.22	.005
Psychological	0.13	0.22	-0.298	0.554	0.05	.554
Physiological	0.34	0.22	-0.088	0.775	0.14	.118

*Note. N* = 172; B = unstandardized coefficient; β = standardised coefficient; CI = confidence interval; LL = lower limit; UL = upper limit. * *p* < .001.

**Table 5.** Symptoms clusters coefficients predicting symptoms impairment during premenstrual phase.

​	95% CI					
Cluster	B	*SE*	LL	UL	β	*p*
Constant	1.09	0.15	0.80	1.38	-	.000*
Pain	1.11	0.19	0.74	1.48	0.48	.000*
Gastrointestinal	-0.00	0.16	-0.31	0.31	0.00	1.000
Discomfort	0.18	0.14	-0.11	0.46	0.08	.222
Psychological	0.12	0.18	-0.24	0.49	0.06	.505
Physiological	0.36	0.21	-0.04	0.77	0.17	.079

*Note. N* = 245; B = unstandardized coefficient; β = standardised coefficient; CI = confidence interval; LL = lower limit; UL = upper limit. * *p* < .001.

## Discussion

This study aimed at addressing the gap in knowledge of a wide range of menstrual symptoms in the German and Austrian populations by investigating their frequency and distress in three phases of the cycle using a validated instrument. Overall, the degree of symptoms interference (i.e., distress) remained modest across all phases. However, while the reported frequency of menstrual and premenstrual symptoms aligns with existing literature, the high prevalence of symptoms during the intermenstrual phase both extends and challenges current understanding of menstrual cycle-related symptoms.

### Symptoms and distress experienced during the menstrual and premenstrual phases

Some of the most reported and somewhat distressful symptoms in this sample – abdominal pain, headache, breast tenderness or swelling, psychological symptoms (i.e., emotional instability, irritability or anger, sadness), as well as fatigue and need for sleep – align with previous large-scale studies conducted both internationally^2,10,13^ and in Austria and Germany.^8,14,22^ Nevertheless, other very frequent and somewhat distressful symptoms, including discomfort with vaginal bleeding, feeling dirty, concentration impairment were ignored in the literature, suggesting that prior symptom reports may be incomplete.

Specifically, discomfort symptoms were the most frequent in the menstrual phase and the second most frequent in the premenstrual phase, while also predicting impairment in the menstrual phase. Breast tenderness and swelling have been also widely investigated in previous large studies and the high frequency is consistent with evidence that cyclical mastalgia – breast pain associated with the menstrual cycle – is closely linked to oestrogen and progesterone fluctuations, and it is most prevalent before menstruation.^29^ However, beyond breast tenderness, the other items included in this clusters reflect menstruating people’s perception of discomfort due to bleeding and negative judgment of their body as “dirty”. Our results are in agreement with data from other Austrian and German samples, which reported symptoms such as “feeling uncomfortable” and “feeling unclean” in 42% and 33% of the participants, respectively.^8,14,22^ These results only emphasise the role of society and media, which, by reinforcing the view of menstruation and menstrual blood as something that must be kept hidden and contained at all costs,^30,31^ may additionally magnify the already perceived functional interference resulting from menstrual symptoms and changes.

However, the frequency of discomfort due to vaginal blood loss outside the menstrual period was oddly very high. Abnormal uterine bleeding (AUB) is a broad term referring to irregularities in menstrual cycle frequency, duration, or flow volume, excluding pregnancy-related causes.^32^ While it is estimated to affect up to one-third of women,^32^ it seems unlikely that such a high proportion of our sample experiences bleeding outside of their menses at this frequency, especially since our sample’s age range excludes individuals in the early post-menarche stage, a group more commonly affected by AUB.^32^ Participants may have interpreted vaginal losses not solely as blood but also as other vaginal discharges, which are commonly experienced throughout the menstrual cycle.^33^ Nonetheless, these findings should be interpreted with caution.

The psychological symptom cluster was mostly reported in the premenstrual, and the second most common and distressful cluster in the menstrual phase. This aligns with evidence supporting the relevance and regularity of psychological symptoms in the premenstrual and menstrual phase in the general population.^2,8,10,13,14^

Interestingly, contrary to the stereotypical view that psychological symptoms such as sadness and irritability being the most distressing during the menstrual cycle, concentration impairment emerged as the most impactful symptom within the psychological cluster during the menstrual phase. However, direct comparisons with previous research in general population are limited. For example, a large study,^10^ which found that concentration difficulties had only a modest impact and low prevalence, investigated this item exclusively in the premenstrual phase. While others^2^ did assess concentration during the bleeding phase, they did not report on the distress associated with it. Still, it seems plausible that attention and concentration would be particularly affected during the most painful phase of the cycle. This explanation aligns with experimental studies of the impact of general^34^ and menstrual pain^35^ on attentional capacities.

When looking at the differences between premenstrual and menstrual distress, the MEDI-Q indicators show that for 28% of the sample all the symptoms experienced during menstruation generate more distress compared to the premenstrual phase. However, it is worth noting that almost the same number of participants (25%) reported all the symptoms experienced during menstruation as present at least with the same level of distress even in the premenstrual phase. This relatively high percentage of symptomatology in the premenstrual phase are inconsistent with that reported in the MEDI-Q scale validation study,^17^ which reports that almost all the sample found the symptoms in the menstrual phase most distressful. This difference may be the result of this paper’s adaptation of the scale (i.e., symptoms distress was investigated in *all* the three stages regardless of the symptoms’ occurrence in the menstrual phase). Moreover, the computation of the added indices (i.e., PS; PSD; IS; ISD) representing the average distress and number of symptoms increasing in distress in the premenstrual phase (vs. menstrual) also suggest that, although the increase in distress is minimal, there are around 2 symptoms, on average, increasing in other phases compared to the menstrual phase. Again, this highlights the importance of a broad assessment across symptoms to capture true variability.

### Symptoms and distress experienced during the intermenstrual phase

The findings extend prior knowledge by suggesting that some cyclic-related symptoms might extend beyond the (pre-)menstrual phases, including the intermenstrual phase. Symptoms were most frequent during the premenstrual and intermenstrual phases, despite the menstrual phase being the most distressing. Although the overall distress levels during the intermenstrual phase were low for most participants (see Table 9 in the Supplementary Materials for a breakdown of the frequency), the high occurrence of intermenstrual symptoms warrants further investigation to determine if these results reflect cycle-related processes or other factors. Indeed our post-hoc analyses revealed that participants with endometriosis reported greater pain and discomfort in the intermenstrual phase than those without gynaecological conditions. Notably, the intermenstrual phase, which follows menstruation and precedes the premenstrual stage, is marked by rapid hormonal fluctuations in ovarian (oestrogen) and pituitary (Luteinising Hormone) activity leading to ovulation.^36^ Beyond ovulation cramps, or “Mittelschmerz” in German,^18^ other symptoms, such as affect changes,^37^ headache^38^ and painful breasts^39^ have also been documented in healthy samples during this phase. Since the days of ovulation were not assessed separately in the present questionnaire, future studies should deliberately focus on the impairment of symptoms experienced during ovulation to shed further light on specific symptoms experienced during this phase of the cycle. Moreover, future research should carefully discern pathological symptoms from healthy changes^40^ which may be experienced in this phase by people with and without gynaecological conditions, beyond endometriosis.

Fatigue was unexpectedly common in the intermenstrual phase; previously, better sleep quality and a longer duration of deep sleep had been reported in the follicular and early luteal phase.^41^ However, given that tiredness and fatigue being relatively recurrent throughout the cycle, they may represent a constant symptom not solely influenced by the cycle and by hormones, but resulting from other factors influencing females more than males, such as sleep disorders and circadian dysregulation.^42,43^ These findings underline the importance of studying symptom fluctuations across the cycle to better disentangle impairments due to the cycle from impairments due to other reasons such as stress due to daily life hassles and (care) responsibilities that especially women face.^44–46^

### Distress and impairments due to the symptoms

Overall, the interference of the symptoms with daily living was relatively low. Although direct comparisons with other studies are challenging due to varying methods of assessing functional interference, our results are consistent with previously reported low levels of distress in cross-cultural (range of symptoms interference mean *M* = 0.30 – 2.41 on a scale from 0 to 10)^10^ and Dutch (range of symptoms interference mean *M*= 3.5 - 4.4 on a scale from 0 to 10)^2^ samples. However, this modest interference contrasts with recent Austrian and German menstrual health reports.^8,22^ In particular, the Austrian report^8^ indicates that over two-thirds of menstruating women experience moderate to severe pain and report a strong to very strong impact of physical and psychosocial symptoms in different areas of life. The discrepancy may stem from the authors not discriminating between menstrual and premenstrual phase and assessing symptoms interference across different areas of life, with sex being the most affected (26%), followed by work life (19%), leisure activities and social (18%), household, unpaid care (17%), and school and education (13%).

Nonetheless, our results indicate distress fluctuation across phases, with the menstrual being the most distressing, aligning with previous research.^2^ Eleven percent of participants scored above the clinical distress cutoff of ≥ 20, slightly below the 16.3% reported in the original validation study,^17^ possibly due to differences in recruitment methods (i.e., clinical populations versus our broader representative sample of the German and Austrian populations).

Pain and discomfort were most strongly related to interference with their daily lives, likely because they reflect direct bodily experiences of the menstrual cycle (e.g., cramps, backaches, tender breasts, and blood loss) linked to hormonal fluctuations. Unlike psychological or gastrointestinal symptoms that may occur independently of the cycle, these physical symptoms are specific to menstruation. Menstrual pain is also shaped by gender identity: for women, it may interfere with socially expected caregiving roles, while for trans and non-binary individuals, it can exacerbate gender dysphoria, highlighting sex-gender incongruence.^47^ In both cases, pain can disrupt identity and daily life. Moreover, pain has also been linked to reduced motivation, impaired goal-directed behaviour,^48^ and lower quality of life.^49^

Symptoms within the discomfort cluster may significantly interfere with daily life, in part due to the internalized social stigma surrounding menstruation.^31^ Fear of visible blood leaks can cause embarrassment and expose one’s menstrual status, influencing how others perceive menstuators.^50^ Additionally, breast tenderness can act as a barrier to physical activity, breast-related discomfort was the second most reported obstacle to exercise in a sample of Mexican women,^51^ highlighting its role in limiting participation in everyday activities.

### Relationships with gynaecological conditions, contraception use, and age

Lastly, our findings indicate that neither age, contraception use, nor gynaecological conditions significantly interacted with menstrual phase in predicting distress levels. This contrasts with previous research reporting age-related effect in dysmenorrhea,^4,8,12,23^ intensity^10^ and frequency^24^ of premenstrual symptoms. However, it is worth noting that these studies focused on either a single symptom (i.e., dysmenorrhea) or examined multiple symptoms restricted to the premenstrual phase. Consequently, such findings may not directly translate to our study, where age was included as a predictor of average distress across *all* symptom clusters and phases. Furthermore, evidence suggests that certain clusters, such as mood symptoms, may remain stable across age groups.^24^ While our sample included a wide age range (18-61), it excluded adolescents, who may exhibit more pronounced patterns of distress.^8^

The lack of effects of gynaecological conditions in predicting changes in distress may be attributed to the dichotomous coding of these variables, which did not account for diagnostic specificity. For example, premenstrual disorders (e.g., PMDD) may explain variations in distress between the premenstrual and menstrual phases, but not between the menstrual and intermenstrual phases. In individuals with these conditions, symptoms typically subside within a few days after the onset of bleeding. However, our post-hoc analyses showed that participants with a diagnosis of endometriosis reported significantly higher levels of discomfort with blood loss and impairment in all phases compared to those without gynaecological conditions. Distressed caused by pain, however, was significantly greater in the endometriosis group only in the premenstrual and intermenstrual phases. Although self-reported diagnosis may have resulted in an underdiagnosed sample, potentially contributing to the lack of observed effects, this remains difficult to verify given the limited prevalence data available for Austria and Germany.^8,52–54^

Lastly, contraceptive use did not interact with phase in predicting changes in distress, aligning with recent findings showing no significant differences in symptomatology between naturally menstruating individuals and those using hormonal contraception.^55^

However, both findings should be interpreted with caution, given the small sample, the self-reported assessment of gynaecological conditions, and the lack of more fine-grained information on diagnostic specificity and diverse hormonal contraceptive formulations/use patterns.

### Limitations and conclusions

This study has several limitations. First, its cross-sectional design and reliance on self-reported measures introduce the possibility of retrospective bias. Second, the menstrual cycle phases were defined using the MEDI-Q cut-off days, which do not account for within-person variation in menstrual phase length and combine hormonally distinct phases^36^ (i.e., late follicular, ovulation, and early luteal) under a single intermenstrual category. Third, symptom frequency in the premenstrual and intermenstrual phases was assessed via a “yes/no” answer using a multidimensional scale that combined ordinal (level of distress) and categorical (occurrence) data. Specifically, participants either reported their level of distress or indicated symptoms absence, which may have inflated symptom frequency by conflating low distress with symptoms presence. Thus, the elevated symptom frequency in these phases should be interpreted with caution. Moreover, although our analytical approach accounted for within-person variance when examining distress changes across phases, the MEDI-Q indices primarily reflect sample-level trends. Given that the menstrual cycle is fundamentally a within-person phenomenon,^36^ our findings offer limited generalisability and insight into individual-level fluctuations in symptom frequency and distress. Future longitudinal studies, using ecological momentary assessments, are needed. Additionally, gynaecological conditions were self-reported, and the sensitivity analyses should be regarded as exploratory and not taken as a basis for firm clinical conclusions. Similarly, the dichotomous coding of both hormonal contraception and gynaecological conditions as between-subject factors in the ANOVAs, and the resulting non-significant effects, should be interpreted with caution. More fine-grained categorical groupings (e.g., specific contraceptive methods or individual gynaecological diagnoses) would have allowed more clinically meaningful conclusions, but this was not feasible due to the small sample subgroup counts and limited statistical power. Furthermore, contrary to the eligibility criterion of 3 cycle per year applied by Vannuccini and colleagues,^17^ we only included participants who had at least one menstrual period in the past 12 months to capture a wider range of menstruating people.

As a result, the MEDI-Q frequency score (A) and A-derived indices may be less interpretable for participants with very infrequent menstruation. Because the exact number of menstrual periods in the past 12 months was not assessed, we were unable to identify such participants or perform sensitivity analyses excluding them. Although, given the relatively young sample and the low prevalence of reported gynaecological conditions, it is plausible that most participants had at least three menstrual periods in the last year, this cannot be verified empirically and should be considered when interpreting the findings, particularly those concerning menstrual specificity.

Finally, the questionnaires were not formally pilot-tested or validated in this population and we did not conduct an a priori power analysis for the outcomes examined in this study, as the sample size was fixed by the design of the broader survey in which this project was embedded. Together these two limitations should be considered when interpreting the findings.

## Conclusions

Indeed, the present findings underline the importance of expanding menstrual symptom research to investigate distressing under-researched symptoms, such as discomfort due to bleeding, concentration impairment, feeling dirty. The notable occurrence of intermenstrual symptoms warrants further investigation to determine whether these reflect cycle-related processes or other factors. This broader approach would enable meaningful between-phase comparisons, currently limited by the predominant focus on the menstrual or premenstrual phases and a restricted symptoms range. Nevertheless, these findings should be interpreted with caution given the small sample, the reliance on self-reported gynaecological conditions, the inclusion of participants who might have menstruated only once in the past year, and the lack of more detailed data on diagnostic specificity and hormonal contraceptive use.

## Supplemental material

Supplemental material - Symptoms and distress across the menstrual cycle in a representative sample of Austrian and German people who menstruate: A cross-sectional study Supplemental material for Symptoms and distress across the menstrual cycle in a representative sample of Austrian and German people who menstruate: A cross-sectional study by Lucia Volpi and Laura M. König by Women's Health.

## Data Availability

The datasets, data preprocessing and analysis codes generated during and/or analysed during the current study are available in the OSF repository: https://osf.io/swupm/files/osfstorage.
