# Supplementary material for: Symptoms and distress across the menstrual cycle in a representative sample of Austrian and German people who menstruate: A cross-sectional study
Source: Womens Health (Lond). 2026 Jul 16;22:17455057261462827. doi: 10.1177/17455057261462827 (PMC13376483; doi:10.1177/17455057261462827)
Supplement: Supplemental material - Symptoms and distress across the menstrual cycle in a representative sample of Austrian and German people who menstruate: A cross-sectional study [file sj-pdf-1-whe-10.1177_17455057261462827.pdf]

Supplementary Material

Table 1

Mean, Standard deviation, Cronbach Alpha values of each symptoms cluster per menstrual phase

Symptoms cluster	Menstrual (Score B)			Premenstrual (Score C)			Intermenstrual (Score D)		
	α	*M*	*SD*	α	*M*	*SD*	α	*M*	*SD*
Pain	.77	1.50	.76	.88	1.05	.85	.91	.95	.87
Gastrointestinal	.81	1.34	.88	.90	.82	.88	.93	.77	.91
Discomfort	.68	1.37	.84	.80	1.05	.91	.87	.97	.96
Psychic & Cognitive	.83	1.43	.80	.94	1.11	.91	.95	.97	.91
Physiological	.61	1.40	.82	.90	1.06	.89	.92	.96	.92
Overall	.91	1.41	.82	.98	1.02	.89	.97	.92	.91

Note. A = Cronbach's Alpha; *M* = mean; *SD* = standard deviation

Table 2

Pain, bleeding-related discomfort, and impairment across menstrual phases in participants with endometriosis vs. those without gynaecological conditions

Variable	Endometriosis		No gynaecological condition		*t*	*p*	Cohen's *d*	95% CI	
	M	*SD*	*M*	*SD*				LL	UL
Pain – menstrual phase	2.12	0.86	1.66	0.95	1.910	.057	0.479	-.015	.972
Pain – premenstrual phase	2.00	0.93	1.25	1.10	2.578	.010	0.686	.160	1.209
Pain – intermenstrual phase	2.07	1.14	1.02	1.07	3.551	.001*	0.978	.430	1.523
Discomfort with blood loss – menstrual phase	2.40	0.83	1.65	0.93	3.001	.003	0.812	.273	1.348
Discomfort with blood loss – premenstrual phase	2.00	1.18	0.95	1.02	3.287	.001	1.016	.402	1.629
Discomfort with blood loss – intermenstrual phase	2.00	1.10	0.84	1.02	3.667	.001*	1.135	.518	1.751
Impairment – menstrual phase	4.50	2.23	2.96	1.85	3.391	.001*	0.824	.343	1.304
Impairment – premenstrual phase	3.83	2.31	2.51	1.84	2.911	.004	0.707	.227	1.186

Note. Endometriosis group n ranged from 18 to 14. No gynaecological condition group n ranged from 289 to 201. *p* = two-sided *p* value; 95% CI for Cohen's *d* estimates; LL = lower limit; UL = upper limit; * $p < .001$.

Table 3

Sample Demographics

	Austria		Germany		Total	
	N	%	*N*	%	*N*	%
Town size						
In a big city	41	31%	72	36%	113	34%
In a medium city	16	12%	42	21%	58	17%
In a small city	12	9%	51	25%	63	19%
In a village	65	49%	37	18%	102	30%
Citizenship						
Yes	131	98%	195	97%	326	97%
No	3	2%	7	3%	10	3%
Migration background						
Yes	22	16%	41	20%	63	19%
No	112	84%	161	80%	273	81%
Identification in minority						
Yes	13	10%	25	12%	38	11%
No	113	84%	168	83%	281	84%
I prefer not to say	8	6%	9	4%	17	5%
Net income						
Less than150€	2	1%	3	1%	5	1%
Da 150€ a 300€	4	3%	1	0%	5	1%
Da 300€ a 500€	2	1%	4	2%	6	2%
Da 500€ a 1.000€	9	7%	9	4%	18	5%
Da 1.000€ a 1.500€	11	8%	18	9%	29	9%
Da 1.500€ a 2.000€	16	12%	23	11%	39	12%
Da 2.000€ a 2.500€	18	13%	24	12%	42	13%
Da 2.500€ a 3.000€	19	14%	39	19%	58	17%
Da 3.000€ a 5.000€	43	32%	59	29%	102	30%
Da 5.000€ a 10.000€	7	5%	21	10%	28	8%
Occupation						
I have a full-time job	48	36%	93	46%	141	42%
I have a part-time job	43	32%	50	25%	93	28%
I am seeking employment	11	8%	15	7%	26	8%
I am studying	22	16%	30	15%	52	15%
I am exclusively a home maker	8	6%	11	5%	19	6%
I am retired	2	1%	3	1%	5	1%
Highest education certificate						
No school-leaving certificate (yet)	0	0%	2	1%	2	1%
Elementary school / secondary school certificate	14	10%	15	7%	29	9%
Secondary school leaving certificate	12	9%	80	40%	92	27%
Completion of polytechnic secondary school	6	4%	2	1%	8	2%
Completion of a (vocational) college	66	49%	19	9%	85	25%
University entrance qualification (Abitur/ Matura)	33	25%	83	41%	116	35%
Other	3	2%	1	0%	4	1%
Highest qualification certificate						
No completed vocational training (yet)	30	22%	47	23%	77	23%
Apprenticeship	25	19%	69	34%	94	28%
Technical secondary school/vocational secondary school	48	36%	18	9%	66	20%
Technical college	10	7%	17	8%	27	8%
University	13	10%	46	23%	59	18%
Doctorate	2	1%	2	1%	4	1%
Other	6	4%	3	1%	9	3%

Table 4

Symptoms absolute frequency during the menstrual phase ordered by distress level (within cluster)

Symptoms	Never Occurred		Less than half of the menses		At least half of the menses		If occurrence, average distress		Mode
	N	% Sample	*N*	% Sample	*N*	% Sample	*M*	*SD*	
**Cluster: Pain**							1.49	0.76	0
Headache	122	36%	114	34%	100	30%	1.76	0.92	0
Abdominal pain	51	15%	111	33%	174	52%	1.69	0.94	2
Muscle or osteoarticular pain	176	52%	82	24%	78	23%	1.58	0.92	0
Pain during sex	266	79%	42	13%	28	8%	1.36	0.93	0
Pain when urinating	282	84%	32	10%	22	7%	1.35	1.00	0
Pain during bowel movement	251	75%	50	15%	35	10%	1.16	0.95	0
**Cluster: gastrointestinal**							1.34	0.88	0
Nausea	234	70%	63	19%	39	12%	1.57	0.96	0
Digestive problems	218	65%	63	19%	55	16%	1.42	0.96	0
Constipation	256	76%	55	16%	25	7%	1.38	0.96	0
Diarrhoea	200	60%	73	22%	63	19%	1.36	0.96	0
**Cluster: discomfort**							1.37	0.84	0
Discomfort due to vaginal bleeding	149	44%	68	20%	119	35%	1.72	0.94	0
Feeling of being dirty	158	47%	67	20%	111	33%	1.47	1.00	0
Breast tenderness or swelling	129	38%	93	28%	114	34%	1.17	0.96	0
**Cluster: Psychic and cognitive**							1.43	0.79	0
Concentration difficulties	219	65%	73	22%	44	13%	1.74	0.91	0
Excessive sadness	151	45%	86	26%	99	29%	1.59	0.94	0
Anxiety	199	59%	76	23%	61	18%	1.58	0.88	0
Irritability or anger	116	35%	110	33%	110	33%	1.56	0.91	0
Emotional instability	113	34%	100	30%	123	37%	1.55	0.91	2
Impulsivity	207	62%	75	22%	54	16%	1.47	0.94	0
**Cluster: physiological**							1.40	0.82	0
Fatigue	117	35%	101	30%	118	35%	1.75	0.94	2
Insomnia	213	63%	77	23%	46	14%	1.72	0.95	0
Increase need for sleep	177	53%	83	25%	76	23%	1.64	0.94	2
Increased appetite	154	46%	87	26%	95	28%	1.42	1.06	0
Decreased sexual drive	180	54%	65	19%	91	27%	1.16	1.01	0
Decreased appetite	259	77%	48	14%	29	9%	1.09	1.04	0

Note. "% sample" represents the percentage of symptoms relative to the sample population (i.e. $N = 336$); M = mean distress level; SD = standard deviation; Mode: 2 = at least half of the menses; 0 = never occurred. N of participants reporting symptoms as “never occurring” corresponds to logical missingness and was excluded for the calculation of mean and SD distress value in the table.

Table 5

Menstrual-phase symptom occurrence and distress ratings, by symptom cluster

**Symptom**	**Distress**									**Symptom does not occur**			**if yes, average distress**		
	No distress			Mild			Moderate - Severe								
	N	% Sample	% Cluster	*N*	% Sample	% Cluster	*N*	% Sample	% Cluster	*N*	% Sample	% Cluster	*M*	SD	Mode
**Cluster: Pain**			5%			17%			21%			57%	1.49	0.76	
Headache	16	5%		74	22%		124	37%		122	36%		1.76	0.92	1
Abdominal pain	26	8%		106	32%		153	46%		51	15%		1.69	0.94	1
Muscle or osteoarticular pain	16	5%		68	20%		76	23%		176	52%		1.58	0.92	1
Pain during sex	12	4%		31	9%		27	8%		266	79%		1.36	0.93	1
Pain when urinating	10	3%		25	7%		19	6%		282	84%		1.35	1.00	1
Pain during bowel evacuation	23	7%		34	10%		28	8%		251	75%		1.16	0.95	1
**Cluster: gastrointestinal**			6%			13%			14%			68%	1.34	0.88	
Nausea	13	4%		39	12%		50	15%		234	70%		1.57	0.96	1
Digestive problems	20	6%		48	14%		50	15%		218	65%		1.42	0.96	1
Constipation	15	4%		32	10%		33	10%		256	76%		1.38	0.96	1
Diarrhoea	27	8%		52	15%		57	17%		200	60%		1.36	0.96	1
**Cluster: discomfort**			10%			23%			24%			43%	1.37	0.84	
Discomfort due to vaginal bleeding	13	4%		76	23%		98	29%		149	44%		1.72	0.94	1
Feeling of being dirty	27	8%		79	24%		72	21%		158	47%		1.47	1.00	1
Breast tenderness or swelling	58	17%		79	24%		70	21%		129	38%		1.17	0.96	1
**Cluster: Psychic and cognitive**			5%			20%			25%			50%	1.43	0.79	
Concentration difficulties	9	3%		40	12%		68	20%		219	65%		1.74	0.91	1*
Excessive sadness	22	7%		69	21%		94	28%		151	45%		1.59	0.94	1
Anxiety	12	4%		57	17%		68	20%		199	59%		1.58	0.88	1
Irritability or anger	24	7%		90	27%		106	32%		116	35%		1.56	0.91	1
Emotional instability	24	7%		94	28%		105	31%		113	34%		1.55	0.91	1
Impulsivity	19	6%		53	16%		57	17%		207	62%		1.47	0.94	1
**Cluster: physiological**			8%			16%			21%			55%	1.40	0.82	
Fatigue	16	5%		82	24%		121	36%		117	35%		1.75	0.94	1
Insomnia	11	3%		44	13%		68	20%		213	63%		1.72	0.95	1
Increase need for sleep	14	4%		62	18%		83	25%		177	53%		1.64	0.94	1
Increased appetite	43	13%		56	17%		83	25%		154	46%		1.42	1.06	1
Decreased sexual drive	47	14%		58	17%		51	15%		180	54%		1.16	1.01	1
Decreased appetite	27	8%		27	8%		23	7%		259	77%		1.09	1.04	1**

Note. "No distress" = symptom is present but rated as not interfering with daily life (interference = 0 on the 0–3 scale). "Mild" = symptom interferes somewhat (interference = 1 on the 0–3 scale). "Moderate - Severe" = symptom interferes moderately or very strongly (interference = 2-3 on the 0–3 scale). "% sample" represents the percentage of participant in the total sample (i.e. N = 336); "% cluster" refers to the percentage of all symptom ratings within that cluster (i.e., N = 336*n. of items); *M* = mean, *SD* = standard deviation, Mode: 1 = symptom somewhat interferes with daily life. 2 = symptom moderately interferes with daily life. *M*, *SD*, and Mode describe the interference ratings (0–3) among participants who reported the symptom. The number of participants reporting symptoms absence (i.e., "Symptom does not occur" in the table) corresponds to logical missingness and was excluded from the calculation of the mean distress scores. * Two modes exist: 1 = mild and 2 = moderate. ** Two modes exist: 1 = mild and 0 = no interference.

Table 6

Menstrual-phase symptom distress prevalence by severity and symptom clusters

Symptom	Any vs. No distress				Moderate-Severe vs. No-Mild distress			
		95%CI				95%CI		
	% Sample	*LL*	*UL*	*p*	% Sample	*LL*	*UL*	*p*
**Cluster: Pain**								
Headache	93%	89%	96%	.000*	58%	51%	64%	.024
Abdominal pain	91%	87%	94%	.000*	54%	48%	59%	.236
Muscle or osteoarticular pain	90%	85%	94%	.000*	48%	40%	55%	.580
Pain during sex	83%	73%	90%	.000*	39%	28%	50%	.073
Pain when urinating	82%	70%	90%	.000*	35%	23%	48%	.041
Pain during bowel evacuation	73%	63%	82%	.000*	33%	24%	43%	.002
**Cluster: gastrointestinal**								
Nausea	87%	80%	93%	.000*	49%	39%	59%	.921
Digestive problems	83%	76%	89%	.000*	42%	34%	51%	.118
Constipation	81%	72%	89%	.000*	41%	31%	52%	.146
Diarrhoea	80%	73%	86%	.000*	42%	34%	50%	.072
**Cluster: discomfort**								
Discomfort due to vaginal bleeding	93%	89%	96%	.000*	52%	45%	60%	.559
Feeling of being dirty	85%	79%	90%	.000*	40%	33%	48%	.013
Breast tenderness or swelling	72%	66%	78%	.000*	34%	28%	40%	.000*
**Cluster: Psychic and cognitive**								
Concentration difficulties	92%	87%	96%	.000*	58%	49%	67%	.096
Excessive sadness	88%	83%	92%	.000*	51%	44%	58%	.883
Anxiety	91%	86%	95%	.000*	50%	41%	58%	1.00
Irritability or anger	89%	85%	93%	.000*	48%	42%	55%	.637
Emotional instability	89%	85%	93%	.000*	47%	41%	54%	.422
Impulsivity	85%	79%	91%	.000*	44%	36%	53%	.218
**Cluster: physiological**								
Fatigue	93%	89%	96%	.000*	55%	49%	62%	.137
Insomnia	91%	85%	95%	.000*	55%	47%	64%	.279
Increase need for sleep	91%	86%	95%	.000*	52%	44%	60%	.634
Increased appetite	76%	70%	82%	.000*	46%	39%	53%	.266
Decreased sexual drive	70%	62%	77%	.000*	33%	26%	40%	.000*
Decreased appetite	65%	54%	75%	.012	30%	20%	41%	.000*

Note. “Any vs. no distress” shows the prevalence of any distress (score ≥ 1) with binomial 95% CIs and p values. “Moderate–severe vs. no–mild distress” shows the prevalence of moderate–severe distress (score ≥ 2) with binomial 95% CIs and p values. CI = confidence interval estimated using Likelihood Ratio; LL = lower limit; UL = upper limit; p = p value of binomial tests. * p = < .001

Table 7

Premenstrual-phase symptom occurrence and distress ratings, by symptom cluster

Symptom	Interference									Symptom does not occur			if yes, average distress		
	No interference			Mild			Moderate - Severe								
	N	% Sample	% Cluster	*N*	% Sample	% Cluster	*N*	% Sample	% Cluster	*N*	% Sample	% Cluster	*M*	SD	Mode
**Cluster: Pain**			33%			17%			19%			31%	1.05	0.85	
Abdominal pain	85	25%		90	27%		116	35%		45	13%		1.32	1.11	1
Headache	74	22%		89	26%		103	31%		70	21%		1.32	1.10	1
Muscle or osteoarticular pain	98	29%		77	23%		68	20%		93	28%		0.99	1.02	0
Pain during sex	125	37%		39	12%		29	9%		143	43%		0.57	0.91	0
Pain during bowel evacuation	144	43%		32	10%		34	10%		126	38%		0.53	0.90	0
Pain when urinating	143	43%		25	7%		31	9%		137	41%		0.52	0.94	0
**Cluster: gastrointestinal**			36%			15%			14%			35%	0.82	0.88	
Digestive problems	120	36%		50	15%		52	15%		114	34%		0.80	1.02	0
Diarrhoea	118	35%		59	18%		49	15%		110	33%		0.80	0.99	0
Nausea	124	37%		52	15%		49	15%		111	33%		0.76	1.00	0
Constipation	127	38%		42	13%		33	10%		134	40%		0.63	0.97	0
**Cluster: discomfort**			31%			23%			21%			26%	1.05	0.91	
Discomfort due to vaginal bleeding	99	29%		79	24%		73	22%		85	25%		1.05	1.07	0
Breast tenderness or swelling	100	30%		90	27%		69	21%		77	23%		1.01	1.03	0
Feeling of being dirty	110	33%		62	18%		68	20%		96	29%		0.98	1.11	0
**Cluster: Psychic and cognitive**			29%			23%			25%			24%	1.11	0.91	
Emotional instability	80	24%		95	28%		103	31%		58	17%		1.25	1.05	1
Irritability or anger	85	25%		89	26%		94	28%		68	20%		1.21	1.07	1
Excessive sadness	95	28%		71	21%		98	29%		72	21%		1.18	1.10	0
Anxiety	104	31%		75	22%		68	20%		89	26%		0.98	1.04	0
Concentration difficulties	109	32%		61	18%		68	20%		98	29%		0.97	1.09	0
Impulsivity	106	32%		67	20%		63	19%		100	30%		0.94	1.05	0
**Cluster: physiological**			32%			19%			20%			29%	1.06	0.89	
Fatigue	84	25%		78	23%		101	30%		73	22%		1.27	1.12	0
Increase need for sleep	93	28%		84	25%		77	23%		82	24%		1.11	1.09	0
Increased appetite	105	31%		62	18%		89	26%		80	24%		1.09	1.11	0
Insomnia	104	31%		71	21%		59	18%		102	30%		0.95	1.06	0
Decreased sexual drive	116	35%		55	16%		48	14%		117	35%		0.77	0.97	0
Decreased appetite	136	40%		33	10%		38	11%		129	38%		0.59	0.93	0

Note. "No distress" = symptom is present but rated as not interfering with daily life (interference = 0 on the 0–3 scale). "Mild" = symptom interferes somewhat (interference = 1 on the 0–3 scale). "Moderate - Severe" = symptom interferes moderately or very strongly (interference = 2-3 on the 0–3 scale). "% sample" represents the percentage of participant in the total sample (i.e. N = 336); "% cluster" refers to the percentage of all symptom ratings within that cluster (i.e., N = 336*n. of items); *M* = mean, *SD* = standard deviation, Mode: 0 = symptoms does not interfere with daily life; 1 = symptom somewhat interferes with daily life. *M*, *SD*, and Mode describe the interference ratings (0–3) among participants who reported the symptom. The number of participants reporting symptoms absence (i.e., "Symptom does not occur" in the table) corresponds to logical missingness and was excluded from the calculation of the mean distress scores.

Table 8

Premenstrual-phase symptom distress prevalence by severity and symptom clusters

Symptom	Any vs. No distress				Moderate-Severe vs. No-Mild distress			
		95%CI				95%CI		
	% Sample	*LL*	*UL*	*p*	% Sample	*LL*	*UL*	*p*
**Cluster: Pain**								
Abdominal pain	71%	65%	76%	.000*	40%	34%	46%	.000*
Headache	72%	67%	77%	.000*	39%	33%	45%	.000*
Muscle or osteoarticular pain	60%	53%	66%	.003	28%	23%	34%	.000*
Pain during sex	35%	29%	42%	.000*	15%	11%	21%	.000*
Pain during bowel evacuation	31%	25%	38%	.000*	16%	12%	22%	.000*
Pain when urinating	28%	22%	35%	.000*	16%	11%	21%	.000*
**Cluster: gastrointestinal**								
Digestive problems	46%	40%	53%	.254	23%	18%	29%	.000*
Diarrhoea	48%	41%	54%	.549	22%	17%	27%	.000*
Nausea	45%	39%	51%	.142	22%	17%	28%	.000*
Constipation	37%	31%	44%	.000*	16%	12%	22%	.000*
**Cluster: discomfort**								
Discomfort due to vaginal bleeding	61%	54%	67%	.001	29%	24%	35%	.000*
Breast tenderness or swelling	61%	55%	67%	.000*	27%	22%	32%	.000*
Feeling of being dirty	54%	48%	60%	.220	28%	23%	34%	.000*
**Cluster: Psychic and cognitive**								
Emotional instability	71%	66%	76%	.000*	37%	32%	43%	.000*
Irritability or anger	68%	63%	74%	.000*	35%	30%	41%	.000*
Excessive sadness	64%	58%	70%	.000*	37%	31%	43%	.000*
Anxiety	58%	52%	64%	.016	28%	22%	33%	.000*
Concentration difficulties	54%	48%	61%	.218	29%	23%	35%	.000*
Impulsivity	55%	49%	61%	.134	27%	21%	33%	.000*
**Cluster: physiological**								
Fatigue	68%	62%	74%	.000*	38%	33%	44%	.000*
Increase need for sleep	63%	57%	69%	.000*	30%	25%	36%	.000*
Increased appetite	59%	53%	65%	.005	35%	29%	41%	.000*
Insomnia	56%	49%	62%	.102	25%	20%	31%	.000*
Decreased sexual drive	47%	41%	54%	.417	22%	17%	28%	.000*
Decreased appetite	34%	28%	41%	.000*	18%	14%	24%	.000*

Note. “Any vs. no distress” shows the prevalence of any distress (score ≥ 1) with binomial 95% CIs and p values. “Moderate–severe vs. no–mild distress” shows the prevalence of moderate–severe distress (score ≥ 2) with binomial 95% CIs and p values. CI = confidence interval estimated using Likelihood Ratio; LL = lower limit; UL = upper limit; *p* = p value of binomial tests. * p = < .001

Table 9

Intermenstrual-phase symptom occurrence and distress ratings, by symptom cluster

Symptom	Distress									Symptom does not occur			if yes, average distress		
	No distress			Mild			Moderate - Severe								
	N	% Sample	% Cluster	*N*	% Sample	% Cluster	*N*	% Sample	% Cluster	*N*	% Sample	% Cluster	*M*	SD	Mode
**Cluster: Pain**			36%			15%			17%			32%	0.95	0.87	
Headache	87	26%		72	21%		96	29%		81	24%		1.18	1.07	0
Abdominal pain	102	30%		77	23%		90	27%		67	20%		1.13	1.1	0
Muscle or osteoarticular pain	114	34%		56	17%		62	18%		104	31%		0.88	1.02	0
Pain during sex	129	38%		35	10%		39	12%		133	40%		0.65	0.99	0
Pain during bowel evacuation	144	43%		35	10%		34	10%		123	37%		0.55	0.92	0
Pain when urinating	140	42%		23	7%		31	9%		142	42%		0.50	0.91	0
**Cluster: gastrointestinal**			38%			13%			12%			36%	0.77	0.91	
Diarrhoea	130	39%		45	13%		45	13%		116	35%		0.72	1.02	0
Digestive problems	126	38%		50	15%		45	13%		115	34%		0.71	0.96	0
Nausea	132	39%		45	13%		41	12%		118	35%		0.64	0.91	0
Constipation	129	38%		38	11%		36	11%		133	40%		0.63	0.97	0
**Cluster: discomfort**			34%			19%			18%			29%	0.97	0.97	
Discomfort due to vaginal bleeding	106	32%		70	21%		61	18%		99	29%		0.97	1.08	0
Feeling of being dirty	112	33%		56	17%		63	19%		105	31%		0.93	1.09	0
Breast tenderness or swelling	123	37%		66	20%		60	18%		87	26%		0.84	1.00	0
**Cluster: Psychic and cognitive**			32%			20%			20%			28%	0.97	0.91	
Emotional instability	101	30%		77	23%		78	23%		80	24%		1.02	1.02	0
Irritability or anger	103	31%		76	23%		72	21%		85	25%		1.00	1.04	0
Excessive sadness	109	32%		67	20%		68	20%		92	27%		0.95	1.05	0
Anxiety	108	32%		65	19%		64	19%		99	29%		0.92	1.03	0
Concentration difficulties	111	33%		62	18%		58	17%		105	31%		0.86	0.99	0
Impulsivity	113	34%		57	17%		56	17%		110	33%		0.85	1.01	0
**Cluster: physiological**			34%			17%			18%			31%	0.96	0.93	
Fatigue	104	31%		71	21%		83	25%		78	23%		1.08	1.10	0
Increase need for sleep	103	31%		71	21%		70	21%		92	27%		1.02	1.08	0
Increased appetite	107	32%		65	19%		69	21%		95	28%		0.96	1.05	0
Insomnia	113	34%		56	17%		61	18%		106	32%		0.89	1.05	0
Decreased sexual drive	124	37%		48	14%		48	14%		116	35%		0.74	0.99	0
Decreased appetite	139	41%		31	9%		37	11%		129	38%		0.57	0.94	0

Note. "No distress" = symptom is present but rated as not interfering with daily life (interference = 0 on the 0–3 scale). "Mild" = symptom interferes somewhat (interference = 1 on the 0–3 scale). "Moderate - Severe" = symptom interferes moderately or very strongly (interference = 2-3 on the 0–3 scale). "% sample" represents the percentage of participant in the total sample (i.e. N = 336); "% cluster" refers to the percentage of all symptom ratings within that cluster (i.e., N = 336*n. of items); *M* = mean, *SD* = standard deviation, Mode: 0 = symptoms does not interfere with daily life; *M*, *SD*, and Mode describe the interference ratings (0–3) among participants who reported the symptom. The number of participants reporting symptoms absence (i.e., "Symptom does not occur" in the table) corresponds to logical missingness and was excluded from the calculation of the mean distress scores.

Table 10

Intermenstrual-phase symptom distress prevalence by severity and symptom clusters

Symptom	Any vs. No distress				Moderate-Severe vs. No-Mild distress			
		95%CI				95%CI		
	% Sample	*LL*	*UL*	*p*	% Sample	*LL*	*UL*	*p*
**Cluster: Pain**								
Headache	66%	60%	72%	.000*	38%	32%	44%	.000*
Abdominal pain	62%	56%	68%	.000*	34%	28%	39%	.000*
Muscle or osteoarticular pain	51%	44%	57%	.844	27%	21%	33%	.000*
Pain during sex	37%	30%	43%	.000*	19%	14%	25%	.000*
Pain during bowel evacuation	32%	26%	39%	.000*	16%	12%	21%	.000*
Pain when urinating	28%	22%	34%	.000*	16%	11%	22%	.000*
**Cluster: gastrointestinal**								
Diarrhoea	41%	35%	48%	.009	21%	16%	26%	.000*
Digestive problems	43%	37%	50%	.044	20%	15%	26%	.000*
Nausea	39%	33%	46%	.002	19%	14%	24%	.000*
Constipation	37%	30%	43%	.000*	18%	13%	23%	.000*
**Cluster: discomfort**								
Discomfort due to vaginal bleeding	55%	49%	62%	.119	26%	21%	32%	.000*
Feeling of being dirty	52%	45%	58%	.693	27%	22%	33%	.000*
Breast tenderness or swelling	51%	44%	57%	.899	24%	19%	30%	.000*
**Cluster: Psychic and cognitive**								
Emotional instability	61%	55%	66%	.000*	31%	25%	36%	.000*
Irritability or anger	59%	53%	65%	.005	29%	23%	35%	.000*
Excessive sadness	55%	49%	62%	.109	28%	23%	34%	.000*
Anxiety	54%	48%	61%	.194	27%	22%	33%	.000*
Concentration difficulties	52%	46%	58%	.599	25%	20%	31%	.000*
Impulsivity	50%	44%	57%	1.000	25%	20%	31%	.000*
**Cluster: physiological**								
Fatigue	60%	54%	66%	.002	32%	27%	38%	.000*
Increase need for sleep	58%	52%	64%	.018	29%	23%	35%	.000*
Increased appetite	56%	49%	62%	.094	29%	23%	35%	.000*
Insomnia	51%	44%	57%	.843	27%	21%	33%	.000*
Decreased sexual drive	44%	37%	50%	.069	22%	17%	28%	.000*
Decreased appetite	33%	27%	39%	.000*	18%	13%	24%	.000*

Note. "Any vs. no distress" shows the prevalence of any distress (score ≥ 1) with binomial 95% CIs and p values. "Moderate–severe vs. no–mild distress" shows the prevalence of moderate–severe distress (score ≥ 2) with binomial 95% CIs and p values. CI = confidence interval estimated using Likelihood Ratio; LL = lower limit; UL = upper limit; *p* = p value of binomial tests. * p = < .001
